# Atypical Retinitis Pigmentosa With Macular Sparing in a Patient With Compound Heterozygous *ABCA4* Variants: A Case Report and Diagnostic Challenge

**DOI:** 10.1002/ccr3.71545

**Published:** 2025-12-25

**Authors:** Na Li, Yalong Dang

**Affiliations:** ^1^ Department of Ophthalmology, Sanmenxia Eye Hospital/Sanmenxia Central Hospital Henan University of Science and Technology Sanmenxia China; ^2^ Henan International Joint Laboratory of Outflow Engineering, Sanmenxia Central Hospital Henan University of Science and Technology Sanmenxia China

**Keywords:** *ABCA4*, macular sparing, retinal degeneration, retinitis pigmentosa, sanger sequencing, whole‐exome sequencing

## Abstract

Inherited retinal dystrophies are a complex group of disorders causing progressive vision loss. The *ABCA4* gene is associated with a wide spectrum of retinopathies, most commonly Stargardt disease, which is characterized by central macular degeneration. Retinitis Pigmentosa (RP) is a less common but recognized *ABCA4*‐associated phenotype, typically involving severe, pan‐retinal degeneration. A 25‐year‐old male presented with a 5‐year history of nyctalopia and progressive peripheral visual field loss. Best‐corrected visual acuity was 1/20 in the right eye and 20/20 in the left eye. Fundus examination revealed pale optic discs, attenuated retinal arteries, and peripheral bone spicule‐like pigment deposits, with notable sparing of the macula. Genetic analysis identified compound heterozygous variants in the *ABCA4* gene: a known pathogenic variant, c.4793C>A (p.Ala1598Asp), inherited from his father, and a novel variant, c.1769A>G (p.Asp590Gly), inherited from his mother. The novel variant was re‐evaluated according to ACMG/AMP guidelines and classified as likely pathogenic based on its absence in population databases, co‐segregation with disease, and high *in silico* prediction scores. This case presents a diagnostic challenge, with a clinical phenotype of macular sparing RP and strong genetic evidence implicating *ABCA4*. These findings expand the potential phenotypic spectrum of *ABCA4*‐retinopathy and underscore the critical role of genetic testing in diagnosing patients with atypical presentations of inherited retinal disease. Further investigation is needed to fully elucidate the pathogenic mechanism of the novel variant and its contribution to this unusual clinical picture.

## Key Clinical Message

This case of retinitis pigmentosa with atypical macular sparing highlights the diagnostic challenges in *ABCA4*‐retinopathy. The identification of compound heterozygous variants, including a novel likely pathogenic variant, suggests the phenotypic spectrum of *ABCA4* may be broader than currently recognized, underscoring the necessity of comprehensive genetic analysis in atypical retinal dystrophies.

## 1 Introduction

Inherited retinal dystrophies (IRDs) are a heterogeneous group of genetic disorders that lead to progressive photoreceptor dysfunction and death, resulting in significant visual loss [1, 2]. Retinitis Pigmentosa (RP) is a primary form of IRD [3], affecting approximately 2.5 million people worldwide (based on a prevalence range of 11.09–26.43 per 100,000) [2] and representing a leading cause of inherited blindness. To date, over 80 genes have been implicated in RP, highlighting its profound genetic heterogeneity [4].

The mutational spectrum associated with *ABCA4* retinopathies includes Stargardt disease, cone‐rod dystrophy, and retinitis pigmentosa, and in several large cohort studies, the *ABCA4* gene is one of the most common causes of hereditary retinal dystrophies [1, 5]. The *ABCA4* gene encodes a retina‐specific ATP‐binding cassette (ABC) transporter located in the outer segment disc membranes of photoreceptors [6]. This protein plays a crucial role in the visual cycle by transporting N‐retinylidene‐phosphatidylethanolamine (N‐Ret‐PE) out of the disc lumen, thereby preventing the formation and accumulation of toxic bisretinoid compounds like A2E in the retinal pigment epithelium (RPE) [7]. Mutations in *ABCA4* are inherited in an autosomal recessive pattern and typically lead to macular‐centric disease.

Here, we present the case of a young man with a clinical phenotype of RP characterized by severe peripheral degeneration but with striking macular sparing. Genetic investigation revealed compound heterozygous variants in the *ABCA4* gene, including one known pathogenic variant and one novel, likely pathogenic variant. This case challenges conventional genotype–phenotype correlations for *ABCA4 retinopathy* and highlights the importance of comprehensive genetic analysis in patients with atypical IRD presentations.

The study adhered to the Declaration of Helsinki and was approved by the Ethics Committee of Sanmenxia Central Hospital (20250121). All participants provided written informed consent.

## 2 Case Presentation

A 25‐year‐old male presented to our hospital with a chief complaint of blurred vision in the right eye and significant night vision difficulties (nyctalopia) for 5 years.

### 2.1 History of Present Illness

Five years prior to presentation, the patient began experiencing a gradual onset of blurred vision in his right eye, accompanied by difficulty seeing in low‐light conditions, particularly while driving at night, and a perceived constriction of his peripheral visual field. These symptoms had worsened over the preceding month, prompting him to seek medical attention. The patient also reported a progressive decline in color vision (dyschromatopsia). There was no history of parental consanguinity.

### 2.2 Physical Examination

**FIGURE 1:**
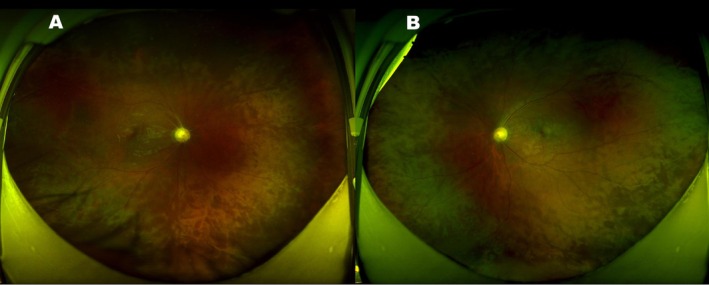
Fundus photographs. Fundus photographs of the right (A) and left (B) eyes. The optic discs appeared pale with clear margins. The retinal arteries were attenuated. The macula was relatively preserved with an attenuated foveal reflex. The peripheral retina showed a mottled appearance with bone spicule‐like pigment deposits.

**FIGURE 2:**
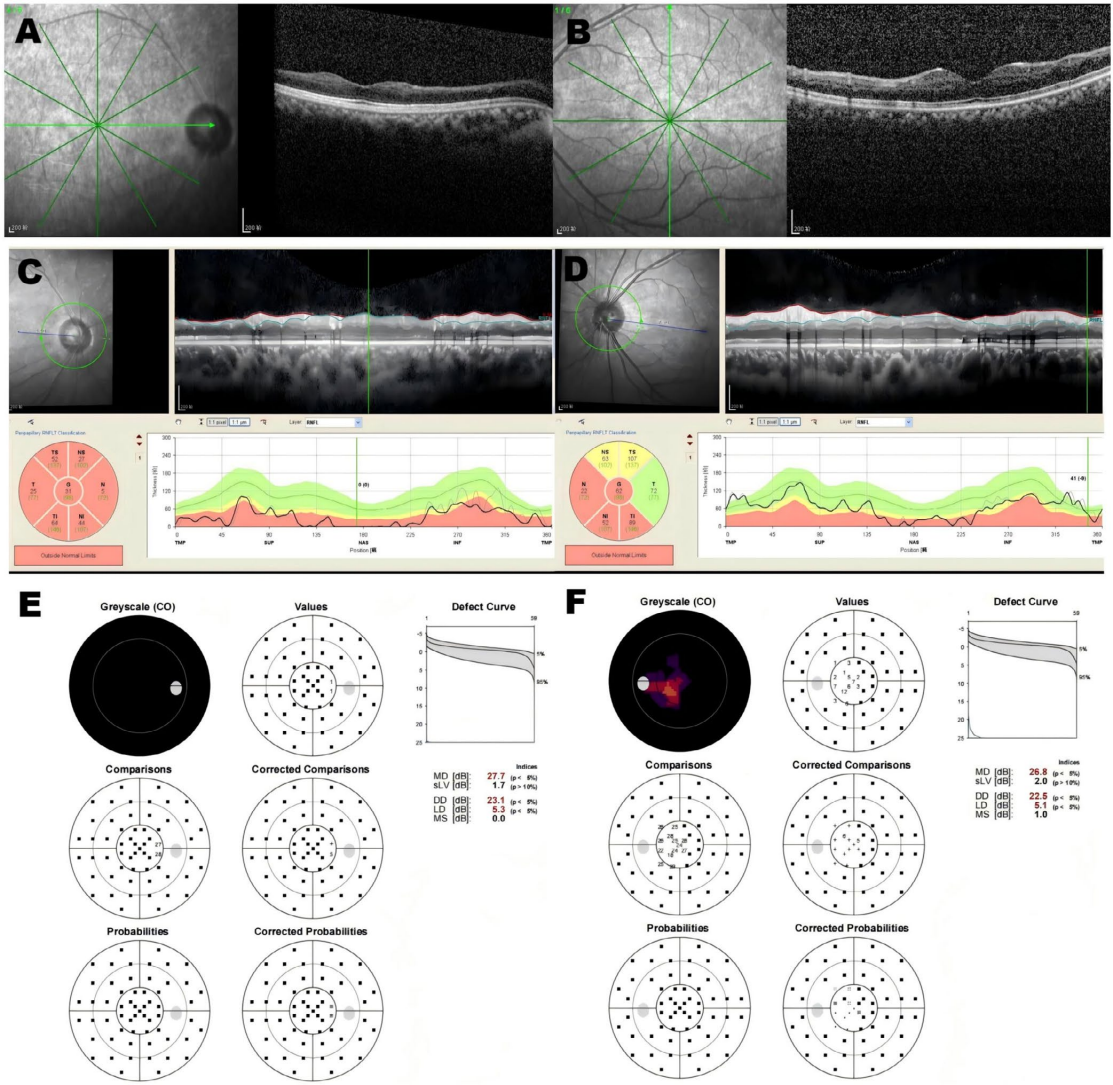
OCT and visual field test. Macular OCT showed ellipsoid zone disruption and outer nuclear layer thinning in both eyes (A, B). Optic nerve OCT imaging showed significant thinning of the retinal nerve fiber layer (C, D). Visual field testing revealed severe constriction to a tubular field of vision in both eyes (E, F).

**FIGURE 3:**
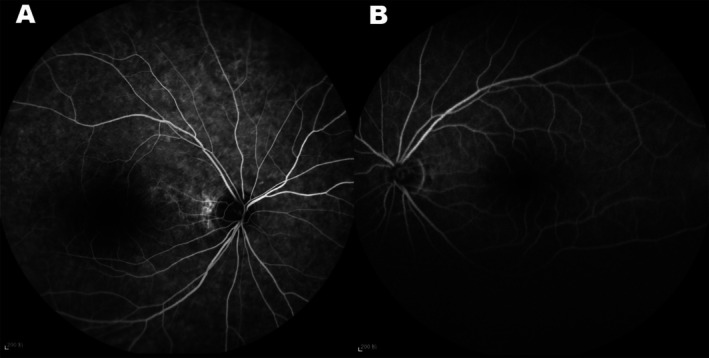
FFA. Late‐phase fundus fluorescein angiography showed hypofluorescence of the optic discs in both eyes, indicative of atrophy.

- Best‐Corrected Visual Acuity (BCVA): The patient's BCVA was 1/20 in the right eye (OD) and 20/20 in the left eye (OS).
- External Examination: Examination of the cornea, lens, and conjunctiva of both eyes was unremarkable. The anterior chamber depth was normal.
- Fundus Examination (Figure 1): In both eyes (OU), the optic discs appeared pale with clear margins. The retinal arteries were markedly attenuated. The macula appeared relatively preserved, though the foveal reflex was attenuated. Subtle yellowish deposits were noted in the perifoveal area and mid‐peripheral retina, along with a mild vitreoretinal interface sheen (Figure S1). The peripheral retina showed a mottled appearance, and classic bone spicule‐like pigment deposits were observed in the mid‐periphery and periphery.
- Optical Coherence Tomography (OCT) (Figure 2A–D): Macular OCT revealed disruption of the ellipsoid zone and thinning of the outer nuclear layer in both eyes, with these changes being more pronounced outside the foveal center. Hyper‐reflective flecks were also present. Optic nerve OCT imaging confirmed significant thinning of the retinal nerve fiber layer in both eyes, with the right eye more severely affected.
- Visual Field (Figure 2E,F): Humphrey visual field testing (24–2 SITA‐Standard) revealed severe, bilateral constriction, consistent with a tubular field of vision. Mean Deviation (MD) was −27.7 dB in the right eye and −26.8 dB in the left eye (*p* < 0.05).
- Fundus Fluorescein Angiography (FFA) (Figure 3): Late‐phase FFA showed hypofluorescence of the optic discs in both eyes, consistent with optic atrophy.

### 2.3 Genetic Testing

**FIGURE 4:**
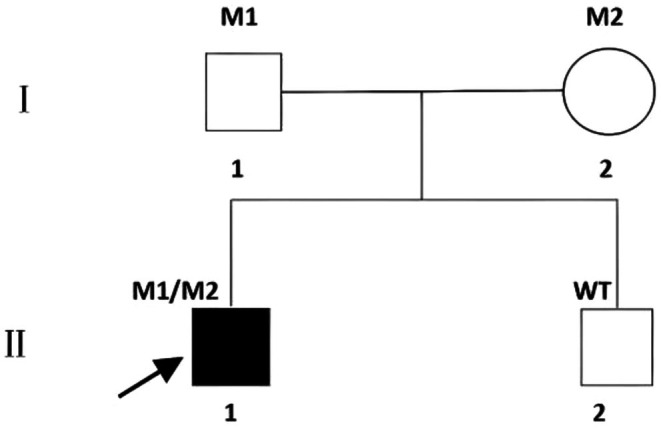
Family pedigree. The pedigree shows the segregation of the *ABCA4* variants. The proband (II:1, arrow) is affected. The father (I:1) is a heterozygous carrier of the c.4793C>A variant (M1). The mother (I:2) is a heterozygous carrier of the c.1769A>G variant (M2). The brother (II:2) is unaffected and carries neither variant (WT).

**FIGURE 5:**
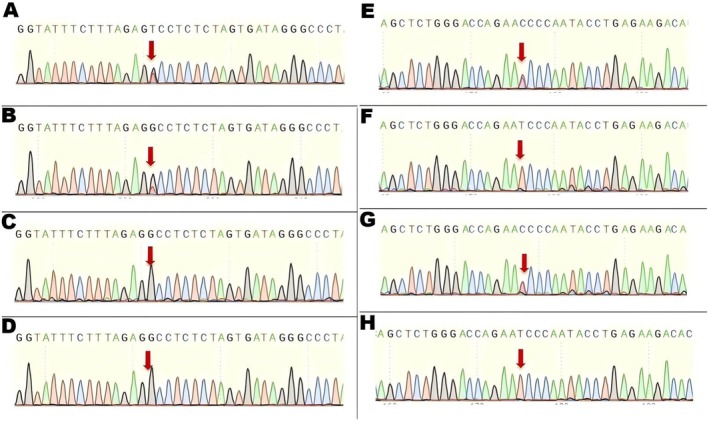
Sanger sequencing. Chromatograms confirm the c.4793C>A and c.1769A>G variants in the patient and his family members, demonstrating inheritance and confirming the variants are in *trans*.

- Methods: Genomic DNA was extracted from peripheral blood leukocytes using the QIAamp DNA Blood Mini Kit (Qiagen, Hilden, Germany) according to the manufacturer's protocol. Whole‐exome sequencing (WES) was performed at the Zhengzhou University Precision Medical Center using the MGI_Exome_Capture_V5 reagent and sequenced on an MGISEQ 2000 platform (BGI) with 150 bp paired‐end reads. Reads were aligned to the human reference genome (GRCh37/hg19). Variant pathogenicity was assessed according to the 2015 ACMG/AMP guidelines. Variants of interest in *ABCA4* were validated and tested for segregation in the patient and his family members (father, mother, and unaffected brother) by Sanger sequencing. Sanger sequencing was performed using BigDye Terminator v3.1 Cycle Sequencing Kit chemistry on an Applied Biosystems 3730xl DNA Analyzer (Thermo Fisher Scientific, Waltham, MA, USA).
- Results: WES identified two heterozygous variants in the *ABCA4* gene (NM_000350.3) in the patient (Table 1).
  1. c.4793C>A (p.Ala1598Asp): This missense variant is classified as pathogenic. It is a well‐documented variant reported in multiple patients with severe retinal dystrophies [8]. It is rare in population databases (gnomAD allele frequency < 0.00001) and affects a functionally important domain of the protein. Its classification is supported by ACMG/AMP criteria PS3, PM1, PM2, and PP3.
  2. c.1769A>G (p.Asp590Gly): This novel missense variant was classified as Likely Pathogenic. It is absent from the gnomAD and ExAC population databases (PM2, moderate). *In silico* analysis predicted a deleterious effect with a high REVEL score of 0.837 (PP3, supporting). The patient's phenotype is highly specific for disease caused by variants in ABCA4 (PP4, supporting). This novel variant has been submitted to the ClinVar database (Accession: SUB15747651).
- Segregation Analysis (Figures 4 and 5): Sanger sequencing confirmed that the patient was compound heterozygous for these two variants. The c.4793C>A variant was inherited from his father, and the novel c.1769A>G variant was inherited from his mother, confirming they are in *trans*. The patient's unaffected brother did not carry either variant (PP1, supporting).

**TABLE 1:** Summary of *ABCA4* variants identified in the patient.

Gene	Transcript	Nucleotide change	Amino acid change	Inheritance	Pathogenicity	Source
*ABCA4*	NM_000350.3	c.4793C>A	p. (Ala1598Asp)	AR	Pathogenic	Father
*ABCA4*	NM_000350.3	c.1769A>G	p. (Asp590Gly)	AR	Variant of Uncertain Significance	Mother

### 2.4 Diagnosis

Based on the clinical findings of progressive nyctalopia and peripheral field loss with bone spicule pigmentation, a diagnosis of retinitis pigmentosa was made. The genetic findings strongly implicate biallelic *ABCA4* variants as the underlying cause.

## 3 Discussion

We report a case of a 25‐year‐old male with a clinical diagnosis of RP and compound heterozygous variants in the *ABCA4* gene. This case is noteworthy due to the striking preservation of the macula, a feature that is highly atypical for *ABCA4 retinopathy* and presents a significant diagnostic challenge.

The *ABCA4* gene is classically associated with Stargardt disease, a juvenile‐onset macular dystrophy where central vision is primarily affected [9]. However, the phenotypic spectrum of *ABCA4* mutations is broad and includes more severe, pan‐retinal conditions such as cone‐rod dystrophy and RP [1]. Biallelic null or severe loss‐of‐function variants are typically associated with these more aggressive phenotypes, which often manifest in the first decade of life [10]. In various cohorts, *ABCA4* mutations have been found to account for 2%–5% of non‐syndromic RP cases, confirming its role in peripheral retinal degenerations [11].

The defining feature of our patient's presentation is the severe peripheral disease coupled with remarkable macular sparing. This topography is contrary to the expected centrifugal pattern of degeneration in *ABCA4* disease, where the macula and posterior pole are most vulnerable [12]. Nevertheless, the concept of regional “sparing” is not entirely foreign to *ABCA4 retinopathy*. Peripapillary sparing is a well‐recognized characteristic, often evident on fundus autofluorescence imaging [13]. Furthermore, relative foveal sparing has been described in some patients, typically those with milder genotypes and later disease onset. Our case could be interpreted as an extreme and rare manifestation on this spectrum of topographical variability, where the degenerative process has, for unknown reasons, predominantly targeted the peripheral retina while leaving the macula functionally and structurally intact.

The genetic evidence provides a strong foundation for implicating *ABCA4*. The paternal variant, c.4793C>A (p.Ala1598Asp), is a known pathogenic allele associated with severe retinal dystrophies, including cone‐rod dystrophy [14]. The maternal variant, c.1769A>G (p.Asp590Gly), is novel. Its reclassification to “Likely Pathogenic” based on rigorous ACMG/AMP criteria—including its absence from population databases (PM2), co‐segregation with disease within the family (PP1), and deleterious *in silico* predictions (PP3)—solidifies the genetic diagnosis. The presence of two pathogenic or likely pathogenic variants in *trans* in a patient with a compatible, albeit atypical, phenotype makes *ABCA4* the most probable causative gene.

This case has several limitations. The primary limitation is the absence of in vitro functional studies to definitively confirm the pathogenicity of the novel p.Asp590Gly variant and to quantify its effect on protein function. Such studies could help explain the unusual phenotype. Additionally, as a single case report, it is not possible to establish a definitive genotype–phenotype correlation; further reports of patients with similar genotypes and phenotypes would be required.

Future therapeutic strategies for *ABCA4*‐retinopathies, such as gene replacement therapy or CRISPR‐based gene editing, are under active investigation [15]. Understanding the full range of clinical phenotypes, including rare presentations like the one described here, is crucial for patient selection and for establishing appropriate outcome measures in future clinical trials.

## 4 Conclusion

We have described a patient with an atypical presentation of macular sparing retinitis pigmentosa associated with compound heterozygous variants in the *ABCA4* gene, including one known pathogenic and one novel, likely pathogenic variant. This case expands the recognized phenotypic variability of *ABCA4*‐retinopathy and highlights the critical importance of comprehensive genetic testing for reaching an accurate diagnosis in patients with IRDs, even when the clinical presentation is atypical. Further research is warranted to understand the molecular mechanisms that lead to such diverse topographies of retinal degeneration from mutations in a single gene.

## Author Contributions

**Na Li:** formal analysis, investigation, visualization, writing – original draft. **Yalong Dang:** conceptualization, formal analysis, investigation, methodology, project administration, supervision, writing – review and editing.

## Funding

This study was supported by the International Science and Technology Co‐operation Program of Henan (Ref. 232102521033), the Medical Education Research Project of Henan (Ref. WJLX2022165), and the Henan Province Medical Science and Technology Key Project (Ref. LHGJ20230956). The funding organizations had no role in the design or conduct of this research.

## Ethics Statement

The study adhered to the Declaration of Helsinki and was approved by the Ethics Committee of Sanmenxia Central Hospital (20250121). Written informed consent was obtained from the patient and participating family members for the publication of their clinical and genetic data, including any accompanying images.

## Conflicts of Interest

The authors declare no conflicts of interest.

## Supporting information


**Figure S1:** Fundus photograph of the posterior pole. The foveal reflex appears attenuated. Subtle yellowish deposits were observed in the perifoveal area and mid‐peripheral retina.

## Data Availability

The raw genetic sequencing data are not publicly available due to patient privacy restrictions but are available from the corresponding author upon reasonable request. The novel variant has been submitted to the ClinVar database (Accession: SUB15747651).
