# Supplementary material for: Atypical Retinitis Pigmentosa With Macular Sparing in a Patient With Compound Heterozygous ABCA4 Variants: A Case Report and Diagnostic Challenge
Source: Clin Case Rep. 2025 Dec 25;14(1):e71545. doi: 10.1002/ccr3.71545 (PMC12740048; doi:10.1002/ccr3.71545)
Supplement: Supplementary file 1 — Figure S1: Fundus photograph of the posterior pole. The foveal reflex appears attenuated. Subtle yellowish deposits were observed in the perifoveal area and mid‐peripheral retina. [file CCR3-14-e71545-s001.docx]

**
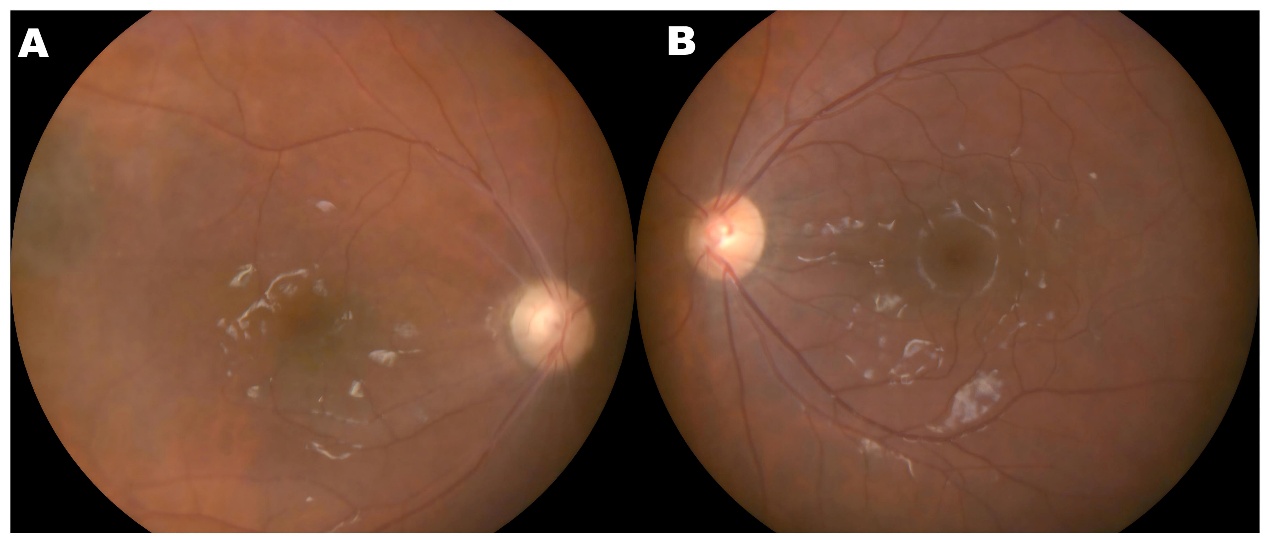
**

**Supple Figure 1. Fundus photograph of the posterior pole.** The foveal reflex appears attenuated. Subtle yellowish deposits were observed in the perifoveal area and mid-peripheral retina.
